# Characterization and outlook of climatic hazards in an agricultural area of Pakistan

**DOI:** 10.1038/s41598-023-36909-4

**Published:** 2023-06-20

**Authors:** Muhammad Tousif Bhatti, Arif A. Anwar, Kashif Hussain

**Affiliations:** 1 International Water Management Institute, 12 Km Multan Road Chowk Thokar Niaz Baig, Lahore, Pakistan; 2 University of Birmingham, Dubai, United Arab Emirates

**Keywords:** Climate-change impacts, Natural hazards

## Abstract

Many dimensions of human life and the environment are vulnerable to anthropogenic climate change and the hazards associated with it. There are several indices and metrics to quantify climate hazards that can inform preparedness and planning at different levels e.g., global, regional, national, and local. This study uses biased corrected climate projections of temperature and precipitation to compute characteristics of potential climate hazards that are pronounced in the Gomal Zam Dam Command Area (GZDCA)— an irrigated agricultural area in Khyber Pakhtunkhwa province of Pakistan. The results answer the question of what the future holds in the GZDCA regarding climate hazards of heatwaves, heavy precipitation, and agricultural drought. The results of heatwaves and agricultural drought present an alarming future and call for immediate actions for preparedness and adaptation. The magnitude of drought indices for the future is correlated with the crop yield response based on AquaCrop model simulations with observed climate data being used as input. This correlation provides insight into the suitability of various drought indices for agricultural drought characterization. The results elaborate on how the yield of wheat crop grown in a typical setting common in the South Asian region respond to the magnitude of drought indices. The findings of this study inform the planning process for changing climate and expected climate hazards in the GZDCA. Analyzing climate hazards for the future at the local level (administrative districts or contiguous agricultural areas) might be a more efficient approach for climate resilience due to its specificity and enhanced focus on the context.

## Introduction

The Intergovernmental Panel on Climate Change (IPCC) emphasizes that human-induced climate change, including more frequent and intense extreme events, has caused widespread adverse impacts and related losses and damages to nature and people, beyond natural climate variability^1^. Scientists^2^ allude that extremes are inevitable as the climate record gets longer, but certain extremes related to heating are becoming more evident. IPCC^1^ has reported on the occurrence of extreme climate events stating an increasing concurrence of heat and drought events which are causing crop production losses and tree mortality. Similarly, hot extremes including heatwaves have intensified in cities. The trends of climate-related extreme events at localized scales are often reported in scientific literature. For example in the United States, extremes of high temperatures have been occurring at a rate twice those of cold extremes^3^. On the other hand, climate change made the South Asian heatwave 30 times more likely^4^.

The term climate hazard usually refers to climate-related physical events or trends or their physical impacts that may cause loss of life, injury, or other health impacts, as well as damage and loss to property, infrastructure, livelihoods, service provision, ecosystems, and environmental resources^5^. Mora et al.^6^ conclude based on a systematic review that with few exceptions, changes in climate hazards have been studied in isolation, whereas impact assessments have commonly focused on specific aspects of human life. Mora et al.^6^ have recently developed a cumulative index that is based on ten climate hazards (warming, precipitation, floods, drought, heatwaves, fires, sea level, storms, changes in natural land cover, and ocean chemistry) and includes six aspects of human systems (health, food, water, infrastructure, economy, and security). They have applied the cumulative index on a global scale and the results are available online as an interactive map.

Due to diversity in geographies and human settlements, some climate hazards and their relevant human system aspects applies more prominently to a particular geographical area than another. The divide between rural and urban agglomeration is well known but a rural or urban area may also exhibit specific ecosystems which call for taking a broader view of relevant climate hazards. Schneider et al.^7^ describe the formal and informal planning instruments that can be used for ecosystem-based adaptation in cities.

As cities are on the frontlines of the global climate emergency, systematic assessments of climate hazards for future and adaptation planning are common for large metropolitans. In the face of climate change, the recently published Lancet report^8^ suggests cities adapt physically to mitigate the negative effects of increasing meteorological extremes. At the beginning of the last decade, there was cognizance of climate change impacts and preparedness by the leadership of large metropolitans. The city of New York has been a prominent case in this regard. The New York City Panel (NPCC) on Climate Change has published its assessment reports^9,10^ that detail climate hazards assessment in the future using several GCMs. According to Rosenzweig et al.^11^ city leaders seem willing and able to take action to protect their cities against these threats and to help make a global difference. The World Mayors Council on Climate Change (WMCCC) was founded in 2005 and the C40 Cities Climate Leadership Group are clear examples of platforms where cities are collaborating to deliver the urgent action needed to confront the climate crisis.

There are several cities in developing countries where human settlement and ecosystems are vulnerable to climate hazards. In many cases, the main source of the community’s livelihood is agriculture and livestock. The lack of systematic assessments of climate change and its impact viewed from various lenses i.e., health food, water, infrastructure, economy, and security, is exacerbating the severity of the challenge.

Pakistan is ranked is amongst the top 8 countries most affected by the impacts of climate-related extreme weather events (storms, floods, heatwaves, etc.) between 2000 to 2019^12^. There is a wealth of literature on climate projections and impacts in Pakistan on a national scale. The literature includes studies conducted by international development agencies^13,14^ and individual researchers. For example, Hussain et al.^15^ have reviewed climate change impacts, adaptation, and mitigation of environmental and natural calamities in Pakistan; and Fahad and Wang^16^ review the climate change, vulnerability, and its impact on rural Pakistan. However, there exists a huge knowledge gap where climate change impacts and resilience is considered at a subnational scale. There are very few cases in Pakistan where climate vulnerability is assessed for planning and preparedness for a city; a prominent case seems to be Islamabad^17^. This shows that climate assessments and planning efforts are commonly skewed towards big cities which sometimes is understandable because of the large population residing there but smaller cities (that sometimes have more vulnerable areas and communities) remain underrepresented. To the best of our knowledge, there is no climate risk assessment available for an agricultural district (in South Asia commonly termed as a ‘command area’ serviced by a water infrastructure like a dam or irrigation canal) in Pakistan. This study has therefore focused on the Gomal Zam Dam Command Area (GZDCA) as it is extended amongst the most underrepresented parts of Pakistan. The area has witnessed large-scale violence and insurrections during the past decade and based on Multidimensional Poverty Index^18^ its constituent districts (i.e. DI Khan and Tank districts) falls in the top 30% of districts in Pakistan (total 114) with the highest poverty prevalence. Huge development investment has been made in last 10 years to develop irrigation infrastructure for improving agriculture and food production in this marginalized area of GZDCA, but the impacts of climate change are making it challenging to achieve the intended dividends of this development investment.

Few studies have reported drought and heatwave characteristics based on the projected climate in South Asia (and its sub-regions)^19,20^. These studies provide useful information about the anticipated heat stress exposure and its future socioeconomic implications. On the other hand, the government meteorological agency in Pakistan also monitors heatwaves and drought and issues advisories, alerts, and weekly/monthly bulletins^21^ based on their analysis at the district scale. In an agricultural area, climate stress affects crop health the most. Previous research typically only investigated the meteorological drought which considers rainfall as the only variable for index calculation and does not include other climate variables. The agricultural drought in contrast to meteorological drought incorporates the characteristics of the crop and agricultural area (in question) by correlating drought responses with the crop yield using a modeling approach.

This article investigates observed relationships between drought and its consequences in the agriculture system and expands on the quantification of selected climate-related hazards in future time slices. The specific results can inform authorities in GZDCA to make planning and preparedness decisions about climate risks however the global audience would find the broad implications of methods more appealing. The methods and analysis used in this paper have wider geographical applicability therefore the specific results are also compared the results with similar studies in other areas around the globe. The agricultural drought indices have mostly been developed and applied in European countries which correlate well with the yield response of wheat cultivated under Mediterranean conditions. Wheat is the second largest crop in South Asia^22^ where the climatic conditions are much different from the Mediterranean countries. This article fills this knowledge gap by calculating agricultural drought indices for the future and assessing their suitability by correlating with the responses of wheat crop yield grown in typical settings in South Asia. The analytical approach in this paper highlights the importance of predicting climate hazards at a local scale i.e., the agricultural district. Planning and preparedness at a local scale are perhaps a smarter approach as it is more context-specific than the climate change planning guidelines available on a wider scale. The application of techniques has not yet been sufficiently tested in the geographic context and setting used in this study. The present study hence provides a scalable methodology to develop a climate outlook for agricultural areas.

## Methods

### The study area

**Figure 1:**
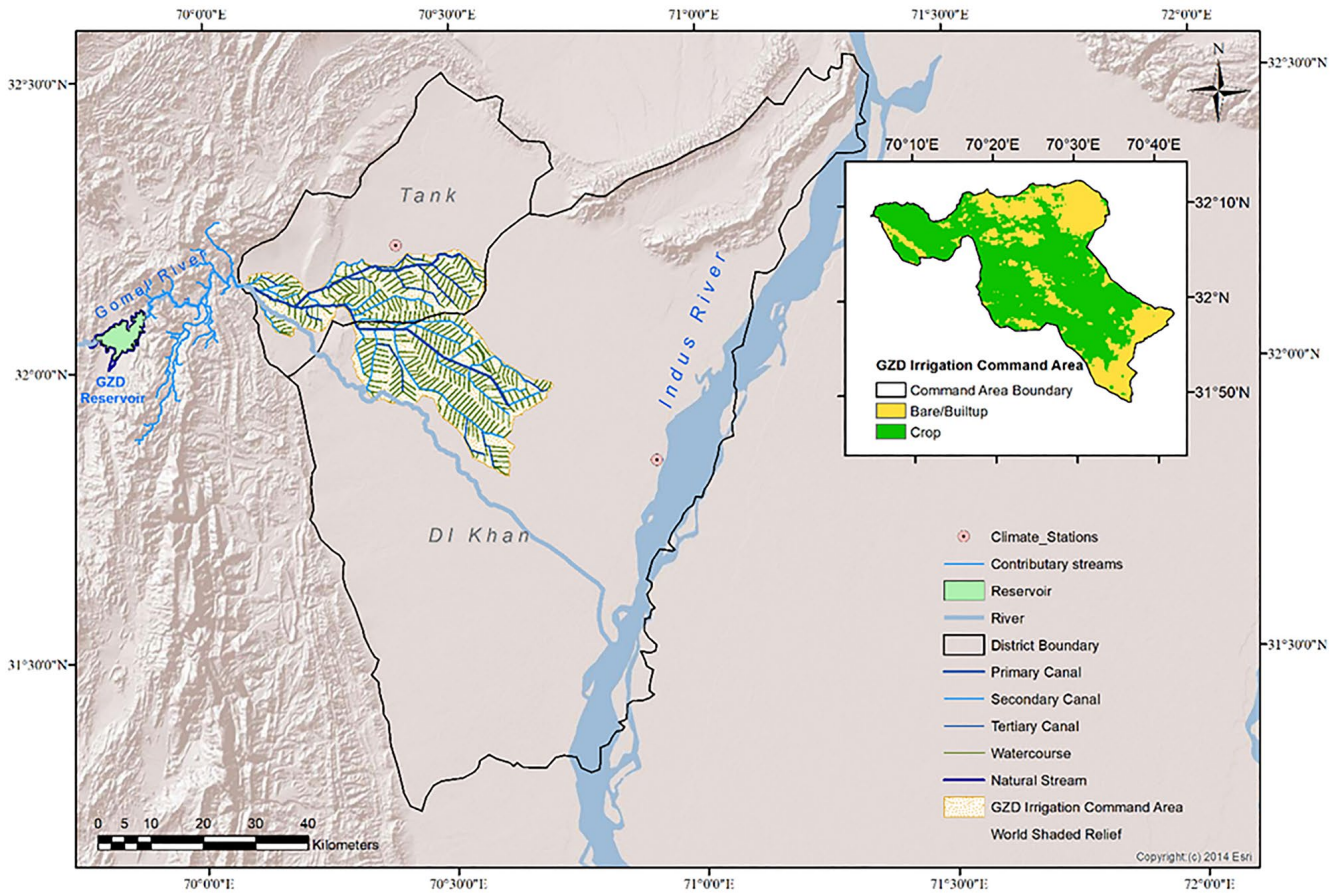
The map of the Gomal Zam Dam Command Area. Basemap credit: Copyright:(c) 2014 Esri, World Shaded Relief.

The climate hazard assessment and evaluation are applied to an area serviced by an irrigation scheme in Pakistan namely Gomal Zam Dam Command Area (GZDCA). The GZDCA is a subset of two adjacent cities/districts in Khyber Pakhtunkhwa province: (i) DI Khan district with a population of 1.6 M (population density = 222 persons/km^2^) out of which 77% resides in areas regarded as rural, and (ii) Tank district having 0.39 M (population density = 233 persons/sq. km) of which 87% lives in rural areas. The total area of GZDCA is 1030 sq. km of which 57% area is in DI Khan and 43% is in the Tank district. In this paper, the climate change analysis uses observed data from two meteorological observatories in GZDCA i.e. DI Khan (31.863, 70.902) and Tank (32.2162, 70.3896) as shown in Fig. 1. The irrigation system comprises a dendritic network of open channels supplying water from the Gomal River to the farms. Figure 1 also shows the important irrigation infrastructures and water delivery systems. The land use with vegetive cover (regarded as cropped area) predominates the built-up/settled area as shown in Fig. 1. Earlier studies^23,24^ have discussed the characteristics of the irrigation system of GZDCA in detail.

### Climate change projection

General circulation models (GCMs) are essential tools for climate studies and are leading means accessible to project future climate and its potential change under various feedbacks from greenhouse gas emissions^25^. However, there has been a growing interest in using Regional Climate Models (RCMs) for climate change impact studies at smaller spatial scales^26^. These RCMS are derived from GCM outputs through dynamical downscaling. The GCM projections are still too coarse (~ 200 km) for explicit impact assessments and computing extreme event indices. Whereas the dynamical approach commonly allows scaling down to about 10–50 km resolution. Large-scale circulations, e.g. shifts in the patterns of monsoonal winds; and local forcings like the topographical, shoreline, and land use heterogeneity, inflect climate change signals at a wide scale (see, e.g.^27–29^). To this end, RCMs are a rational choice particularly when the research motivation is not to understand global climate patterns and trends for which GCMs are the obvious option.

RCMs are high-resolution models able to provide a more realistic representation of important surface heterogeneities (such as topography, coastlines, and land surface characteristics) and mesoscale atmospheric processes^26^. The Coordinated Regional Climate Downscaling Experiment (CORDEX) initiative was the first international program providing a common framework to simulate both historical and future climate at the regional level, under different Representative Concentration Pathways (RCPs)^30^, and over different domains which cover all the land areas. More specifically, it provides climate data simulated by an ensemble of RCMs developed by several research centers all over the world that are forced by GCMs from the Coupled Model Intercomparison Project phase 5 (CMIP5)^31^. Peres et al.^26^ have reviewed 14 studies that incorporated CORDEX RCMs in various regions and listed their key findings. Overall, these studies show that CORDEX RCMs can reproduce the most important climatic features at regional scales, but important biases remain, especially regarding precipitation or climate extremes (depending upon the model, region/sub-region, choices in model configuration, internal variability, and uncertainties of the observational reference data themselves). Peres et al.^26^ draw a similar conclusion based on their analysis to compare the skill of 19 EURO-CORDEX RCMs in reproducing the annual and seasonal temperature and precipitation regime as well as several drought features. The key findings from the analysis have been that the model combinations can simulate temperature better than precipitation, even though important biases do exist in both variables. Models which are reliable in simulating precipitation may not be the same concerning temperature.

In this research, the outputs of several RCMs from the CORDEX-South Asia framework have been used. Concerning the limitations discussed in the aforecited literature, this study has incorporated: a combination of several RCMs to address the issue of inter variability of reliable results of RCMs for different variables; and applied a statistical downscaling approach to improving spatial resolution as well as to minimize biases. The rationale behind the statistical downscaling of CORDEX RCM results is to capture local-scale variations in climate variables that are not well represented in the RCM outputs and resultantly can limit their usefulness for decision-making at local scales. For example, if a stakeholder is interested in understanding the impacts of climate change on a specific watershed or crop production system, the coarse resolution of the RCM outputs may not provide enough detail to make informed decisions. Ahmad and Rasul^32^ expand on the usefulness of the statistical downscaling technique stating that “Emerging statistical downscaling approaches however rely upon provision of long-term datasets of predictor and predictant variables that generate statistically significant relationships. If these provisions are met, then statistical downscaling approaches may present better contingencies for downscaling to precise locations or regions where dynamical downscaling outcomes are uncertain”.

The CORDEX RCM data was acquired from the freely available resource^33^ developed by Copernicus—European Union's Earth observation program. Table 1 provides features of the climate data which includes the list of Global and Regional Climate Models (RCMs) and their spatial and temporal resolution.

**Table 1:** Data for climate projections.

Feature	Description	
Domain	South Asia	
Global climate models (GCM)	CanESM2	Canadian Centre for Climate Modelling and Analysis, Canada
	ESM-LR	Max Planck Institute for Meteorology, Germany
	MIROC5	Japan Agency for Marine-Earth Science and Technology, Japan
	Mk3.6.0	Commonwealth Scientific and Industrial Research Organization; Queensland Climate Change Centre of Excellence, Australia
	EC-EARTH	Irish Centre for High-End Computing, Ireland
Regional climate models (RCM)	RegCM4-4	Indian Institute of Tropical Meteorology, India
	RCA4	Swedish Meteorological and Hydrological Institute, Sweden
	REMO2009	Max Planck Institute for Meteorology, Climate Service Center Germany
Variant label	r1i1p1	
Data type	Gridded	
Horizontal resolution	0.44° × 0.44°	
Temporal resolution	Daily	
Variables (predictands)	Precipitation Minimum temperature Maximum temperature	
Historic period	The CORDEX experiment covers a period for which modern climate observations exist. Boundary conditions are provided by GCMs. These experiments, which follow the observed changes in climate forcing, show how the RCMs perform for the past climate when forced by GCMs and can be used as a reference period for comparison with scenario runs for the future. The period covered is typically 1950–2005. This study uses a historic period of 1980–2005	
Scenario	Ensemble of CORDEX climate projection experiments using RCP (Representative Concentration Pathways) forcing scenarios. This study uses two scenarios: RCP 4.5 and 8.5 providing different pathways of future climate forcing. Boundary conditions are provided by GCMs. The period covered is 2006–2100	

Using a composite observed temperature and precipitation record (derived by averaging the daily data over the two GZDCA stations) results from 5 Regional Climate Models (RCMs) were bias-corrected to project variables (predictands) for future time slices following the methods of Teutschbein and Seibert^34^. Climate Model data for hydrologic modeling (CMhyd) tool^35^ was used to perform bias correction. This tool is widely applied in many hydrological modeling studies^36–38^. The mean and standard deviation of a given variable was used to adjust the model distribution against the target observed distribution.

Observed data of minimum and maximum temperature and precipitation is acquired from two climate stations (observatories) in GZDCA namely DI Khan and Tank. The base period is considered as 1980–2005 where the observed data is used for training the CMhyd model and generating time series for future bias correction of RCMs. The future data is projected from 2006 to 2100. The results are aggregated in three future time slices: Near future or 2020s (2011–2040); Middle of the century or 2050s (2041–2070); and End of the century or 2080s (2071–2100).

### Climate hazards

As climate varies or changes, several direct influences alter precipitation amount, intensity, frequency, and type^39,40^. Warming accelerates land-surface drying as heat goes into the evaporation of moisture, and this increases the potential incidence and severity of droughts, which have been observed in many places worldwide^41^.

An important work to design the analysis for climate hazards has been the NPCC3 report^9^ which defines the climate hazards and their quantification measures: ‘*A climate hazard is a weather or climate state such as a heat wave, flood, high wind, heavy rain, ice, snow, or drought that can cause harm and damage to people, property, infrastructure, land, and ecosystems. Climate hazards can be expressed in quantified measures, such as flood height in feet, wind speed in miles per hour, and inches of rain, ice, or snowfall that are reached or exceeded in a given period of time’.* This paper discusses selected climate hazards that are prominent in GZDCA. The selection of climate hazards is also in line with the results of Mora et al.^6^ for GZDCA. In the subsequent sub-sections, the methodology to analyze these climate hazards is elaborated. To detect an extreme climate event in future timesteps, bias-corrected statistical downscaled data using CMhyd is used as explained in Climate Change Projection section.

### Heatwaves

Heatwave is an important type of extreme climate event that has been described by several attributes and combinations which constitute various event typologies. There is no universal definition for the conditions that constitute a heatwave. Shafiei Shiva et al.^42^ have collated various definitions from literature and concluded that despite the diversity in the definitions, in most studies four main properties are used to describe the impacts of heat waves i.e. frequency, intensity, duration, and timing^43–45^. Sharma et al.^46^ have reviewed the literature to account for the health effects of heatwaves in the South Asian region. According to their review, heatwaves have not been defined explicitly, and there are many definitions of heatwaves for countries in the South Asian region. It is imperative to state, that many indices are available in the literature that explain different heatwave characteristics e.g., some indices were suggested by the Expert Team on Climate Change Detection and Indices. A recent study^47^ has used these indices to explain the heatwave characteristics and future heat exposure to the population in Africa. Importantly, this study^47^ also reports that there is no standard definition of a heatwave and adopts previous researchers' definitions.

Heatwaves are a notable cause of weather‐related human hospitalization and mortality in many parts of the world^48–52^. The heatwaves also seriously impair agriculture, and water distribution systems particularly when accompanied by meteorological droughts, heat waves can significantly decrease crop yield^53^ and ecology^54^. Many plants are sensitive to extended elevated temperatures^55^. Furthermore, heat waves stress power grids and water distribution systems due to the extra consumption of power for cooling and drinking water^56–58^.

The absence of a universal definition of heatwaves calls for adopting a definition for analyzing heatwaves in this study. This brings the authors to adopt the simplified version of the definition by Ref.^48^ which is “Heat waves are defined as ≥ 2 days with temperature ≥ 95th percentile for the base period”. This definition has been adopted in many studies^9,59,60^. To calculate the properties of heatwave events that qualify the adopted definition, the R code by Shafiei Shiva^61^ is modified. The definitions of heatwave properties used in this paper are similar to those used by Shafiei Shiva et al.^42^:

- Number of hot days (Days): A hot day is a day with an average daily temperature higher than the threshold.
- Frequency of heatwaves: Number of independent heatwaves in each calendar year.
- Duration of heatwaves (Total): The cumulative length of all heat waves in each calendar year.
- Longest heat wave event (Longest): The longest heat wave event in each calendar year.
- The severity of heatwaves: Total heat wave severity is the cumulative value of daily average temperature above the defined threshold as expressed in Eq. (1).
  1 $$Severity\, of\, Heatwaves\,=\,\sum_{i=1}^{i=m}({T}_{avg,day\, i}-{Threshold}_{avg})$$

Where ${T}_{avg,day \,i}$ is the average temperature of ${day}_{i}$ during heatwave based on the adopted definition. ${Threshold}_{avg}$ is the threshold for average daily temperature as per the adopted definition. $m$ is the total length of days during heatwaves.

### Heavy precipitation

Precipitation is the major element among the meteorological variables that affect life and civilization most directly because its extreme variation could cause significant impacts on both human society and the natural environment^62^. However, the nature and magnitude of damages due to floods triggered by precipitation extremes (intensity and frequency) vary with location and largely depend on local preparedness. Davenport et al.^63^ link the flood damages resulting from heavy precipitation to the cost of climate change and analyze more than 6600 reports of state-level flood damages to quantify the historical relationship between precipitation and flood damages in the United States. They have concluded that historic change in precipitation (1988–2017) is the reason for 36% of flood damages in the US.

Regarding GZDCA, very little systematic documentation is available on damages due to flash floods. Often the information about flood damages is provided by Provincial Disaster Management Authorities (PDMA) and local media^64^. Ten flood streams in the Tank district receive flash floods during monsoon season because of heavy precipitation. The main source of flash floods is the hill torrents in the catchment just upstream of GZDCA. These flash floods in the recent past have resulted in the loss of human lives and livestock, breaching of canal sections, inundation of agricultural and residential areas, and economic damage to crops. DI Khan is relatively less vulnerable to flash floods than Tank due to the nature of the terrain.

### Drought indices

Drought is a climate extreme and hazard. It is a recurrent phenomenon with severe impacts in many sectors related to the environment and human life^65^. Many studies have raised the need for efficient drought characterization and monitoring for proactive drought management, while efforts for drought forecasting have also been made^66–71^. Drought characterization is commonly expressed as drought indices.

The World Meteorological Organization (WMO) has published a handbook^72^ that compiles numerous drought indices developed over time. The Standardized Precipitation Index (SPI) is perhaps the most widely used drought index which is the accepted standard worldwide as recommended by WMO^73^. A rather recently developed index is the Reconnaissance Drought Index (RDI)^71,74^. Drought is considered a major natural hazard, adversely affecting agricultural systems and posing problems to food security and subsequent economic and social impacts^75–77^. Therefore, many agricultural variants of drought indices have recently developed that generally incorporate the substitution of total precipitation by effective precipitation and include Potential Evapo Transpiration (PET) in the calculation. Agricultural Standardized Precipitation Index (aSPI)^65^ and Effective Reconnaissance Drought Index (eRDI)^78^ are examples of agricultural drought indices.

This study calculates SPI, RDI, and their variants for agricultural drought (i.e. aSPI and eRDI). We use a specialized software package named Drought Indices Calculator (DrinC ver. 1.7)^79^, which has been established to provide a simple interface for the estimation of drought indices. The readers are referred to the user manual of DrinC to understand the data requirement and formats for the calculation of selected drought indices in this study. In our calculations we have selected the Hargreaves method for the estimation of PET; preferred Gamma distribution and USDA (CROPWAT version) as a method for effective precipitation estimation. Generally, a negative value of SPI or RDI value denotes the prevalence of a drought year while a positive value indicates a wet year. This study adopts the drought classification criteria based on eRDI from Tigkas et al.^78^.

### Crop modeling

The Government of Pakistan^80^ considers wheat as a strategic crop, the shortfall in wheat production leads to political uncertainty, significant drainage of foreign reserves, a rise in prices of wheat flour, and pocket shortages in vulnerable areas. Being the main staple crop in Pakistan, wheat ensures the food security of the country. It has grown over 22 million acres and accounts for 7.8 percent of the value-added in agriculture and 1.8 percent of GDP. Self-sufficiency in wheat has been an objective of every Government and thus always challenges agriculture experts and policymakers.

Drought is one of the main factors that impact crop yields but determining the effect of various drought types on crop yields requires observation data. While many studies allude to the interrelationship between the severity of droughts and crop yields in the context of crops in Pakistan, four recent studies^81–84^ commonly report SPI and SPIE indices for the past two decades using both satellite and ground-observed meteorological data sets. The yield estimation and statistical approaches however differ amongst these studies e.g. Refs.^81–83^ rely on government figures for selected crops as reported by the government agency at district scales; while^84^ applies a yield sensitivity index to analyze crop yield sensitivity and empirical relationships to estimate yield losses. All four studies have commonly inferred that the drought indices strongly correlate with yield variability at different stages of crops. In our analysis, we have gone a step further by calculating the drought index (which has the strongest correlation with yield loss) using projected data.

The crop modeling in this paper is focused on the wheat crop due to its enormous importance and emphasis on the government’s policy. In this study, the water-driven simulation crop model AquaCrop is used, considering a winter wheat crop grown in a typical irrigated area in Pakistan. For detailed information on the AquaCrop model and on the crop yield simulation approach, the reader may refer to the studies^65,71,85,86^. It is noted that winter wheat in GZDCA is sown in November and harvested at the end of April which is a typical wheat crop season in South Asia. The input parameters for AquaCrop were mostly adopted from^87^ except for soil and irrigation application information that is based on observed practices in GZDCA. The model simulations were performed on a yearly time step using daily observed data of precipitation and temperature in DI Khan for the period of 1980 to 2014, while other inputs to the model were not altered in each run. The simulated results (i.e. dry yield; ${Y}_{dry}$ in ton/ha) were compared with the average yield of wheat crop in DI Khan as reported by the government agency^88^ to calculate model efficiency. However, the observed data was not available for the entire simulation period therefore comparison was made only for those years with the available data. Annual yield loss is calculated as given in Eq. (2):

2 $${Y}_{loss,\, i}=\left(\frac{{Y}_{dry,\, i-}{Y}_{pot, \,i}}{{Y}_{pot, \,i}}\right)\times 100$$

where: ${Y}_{loss}$ =Annual Yield Loss (%) in ${i}^{th}$ year. ${Y}_{dry}$ = Dry Yield is the simulated yield of wheat from AquaCrop model in ${i}^{th}$ year. ${Y}_{pot}$ = Potential Yield in the ${i}^{th}$ year. The government agency reports potential yield for different wheat varieties. This study has adopted average value for the varieties grown in the study area.

### Ethics approval and consent to participate

The submitted work is original, and it has not been published elsewhere in any form or language. The manuscript is not under simultaneous consideration in any other journal.

## Results and discussion

### Climate outlook

**Figure 2:**
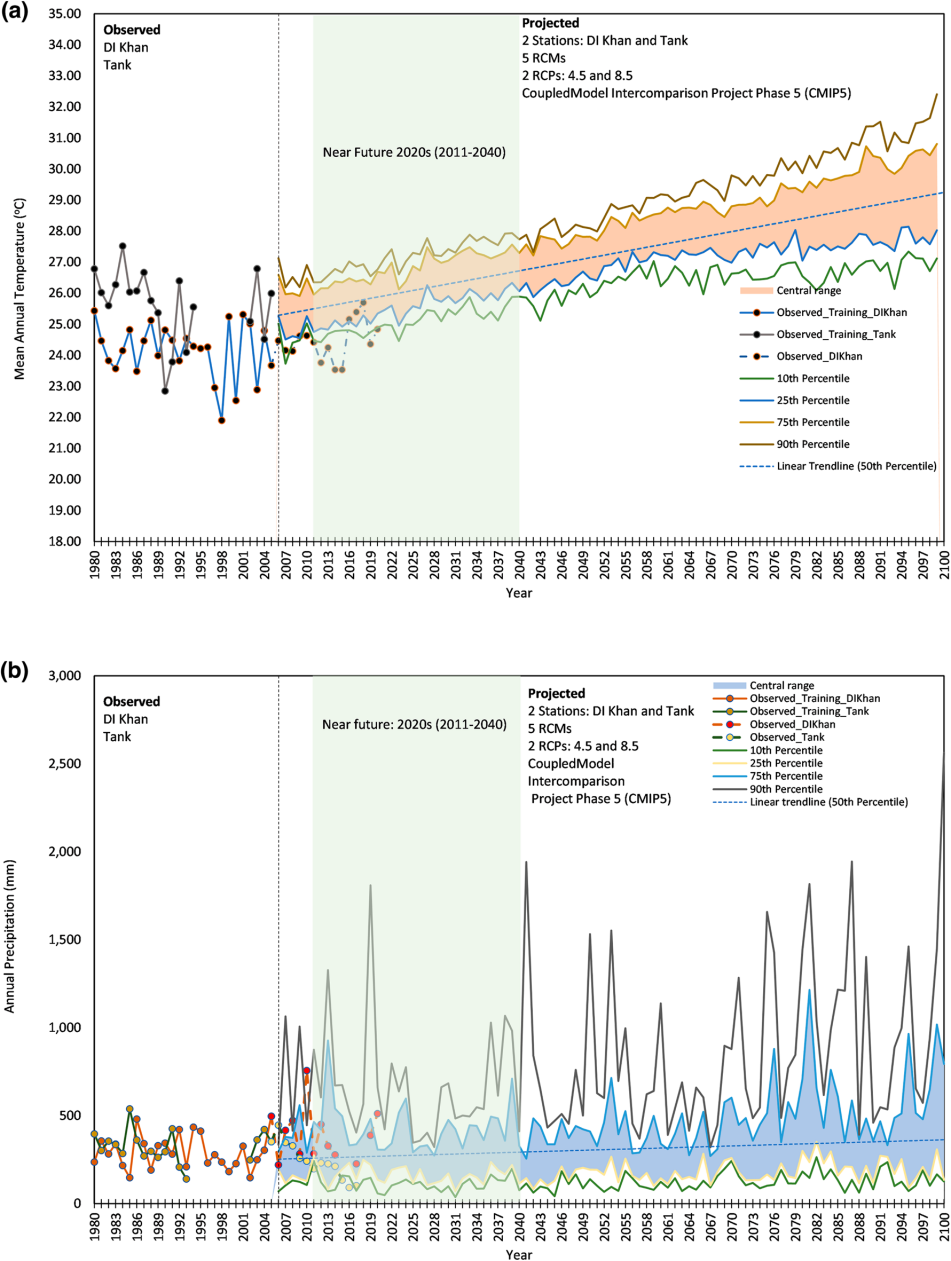
Observations at Gomal Zam Dam Command Area (1980–2005) compared to the future time slice for (**a**) average annual temperature and (**b**) average annual precipitation. Colored lines represent the 10th, 25th, 75th, and 90th percentiles of model projections across RCPs 4.5 and 8.5 for 5RCMs. Shading shows the central range of projections between the 25th and 75th percentiles. Vertical dotted lines represent the range of the observed and projected time slice. Observed data are from the Pakistan Meteorological Department (for DI Khan) and Water and Power Development Authority (for Tank Station), and climate projections are from the Coupled Model Intercomparison Project Phase 5 (CMIP5). These comparisons should be viewed with caution because of the role that natural variation plays in the short term.

Figure 2a shows the annually averaged mean daily observed temperature from two climate stations in GZDCA and the future projections of mean temperature that are based on bias correction applied to 5 RCMs considering two climate pathways (4.5 and 8.5). Observed training data for the base period of 1980–2005 is shown as continuous lines whereas observed data beyond the base period is shown with dashed lines which may be compared with the projected temperature. Recent temperature data from the climate station Tank was not available therefore it is not shown in Fig. 2a.

The future projections in Fig. 2a are shown as the 10th, 25th, 75th, and 90th percentiles of the results from 5 selected RCMs and two climate pathways. The region between the 25th and 75th percentile is shaded and mentioned as the central region. A trend line is also plotted based on the 50th percentile to denote the trend. The trend line (R^2^ = 0.95) shows that GZDCA would experience warming in average temperature conditions by 0.38 °C per decade. The shaded vertical region highlights a time slice of the 2020s comprising the near future that is more important concerning planning and preparedness.

Figure 2b shows the annual precipitation and follows the same plotting scheme as used for mean temperature in Fig. 2a. Observed precipitation data beyond the base period aligns well with the projected data. However, the R^2^ of the trendline for the 50th percentile is very weak (R^2^ = 0.17) that shows no clear trend of increase or decrease in precipitation. Therefore, it is worthwhile to analyze the daily precipitation data for the detection of rainfall events above certain thresholds.

### Precipitation and flooding

Table 2 reports the number of days with rainfall greater than thresholds ranging from 12.7 mm to 50.8 mm. The rainfall in GZDCA mostly happens during the monsoon period (Jul-Sep). Rainfall events up to 25 mm are perceived by farmers as beneficial for agriculture. Anecdotally, rainfall events greater than 25 mm triggers hill torrents and landslides in the catchment area just above the GZDCA. The flash flood in the Gomal River also brings huge amounts of sediments that in case enter the irrigation system reduce the flow carrying capacity of canals and call for dredging. Interestingly, the number of projected rainy days of high intensity is not much different for the selected pathways (4.5 and 8.5). Low-intensity rainfall is more frequently expected when RCP 8.5 is considered. The rainfall days greater than the selected thresholds are disaggregated in three-time slices. The results for the near future are more important for planning and preparedness.

**Table 2:** Predicted rainy days for GZDCA above various thresholds in future time steps.

Rainy days	Baseline DIKhan and tank	DIKhan		Tank	
	(1980–2005)	RCP 4.5	RCP 8.5	RCP 4.5	RCP 8.5
**Near future 2020s (2011–2040)**					
Number of days rainfall > 12.7 mm (0.5 inch)	7.00	0.00	0.00	0.07	9.83
Number of days rainfall > 25.4 mm (1 inch)	2.62	2.17	1.27	2.47	1.97
Number of days rainfall > 38.1 mm (1.5 inch)	1.27	0.97	0.90	1.37	1.03
Number of days rainfall > 50.8 mm (2 inch)	0.46	0.67	0.43	0.90	0.57
**Middle of the century 2040s: (2041–2070)**					
Number of days rainfall > 12.7 mm (0.5 inch)	7.00	0.00	0.00	0.13	10.73
Number of days rainfall > 25.4 mm (1 inch)	2.62	2.20	2.87	2.47	2.57
Number of days rainfall > 38.1 mm (1.5 inch)	1.27	1.27	1.50	1.63	1.43
Number of days rainfall > 50.8 mm (2 inch)	0.46	0.57	0.93	0.93	1.07
**End of the century 2080s: (2071–2100)**					
Number of days rainfall > 12.7 mm (0.5 inch)	7.00	0.00	0.00	0.33	12.73
Number of days rainfall > 25.4 mm (1 inch)	2.62	3.50	4.77	3.10	3.53
Number of days rainfall > 38.1 mm (1.5 inch)	1.27	1.67	2.83	1.97	2.53
Number of days rainfall > 50.8 mm (2 inch)	0.46	1.07	2.07	1.57	1.87

In Fig. 2b the observed precipitation in the 2020s leaned toward high future estimates i.e., 90^th^ percentile. Looking at the RCP8.5 in Table 2 the number of rainy days with all low thresholds (< 25.4 mm) would be greater than the baseline average. A rainfall event of around 50 mm in a day can result in floods with devastating impacts on GZDCA.

**Figure 3:**
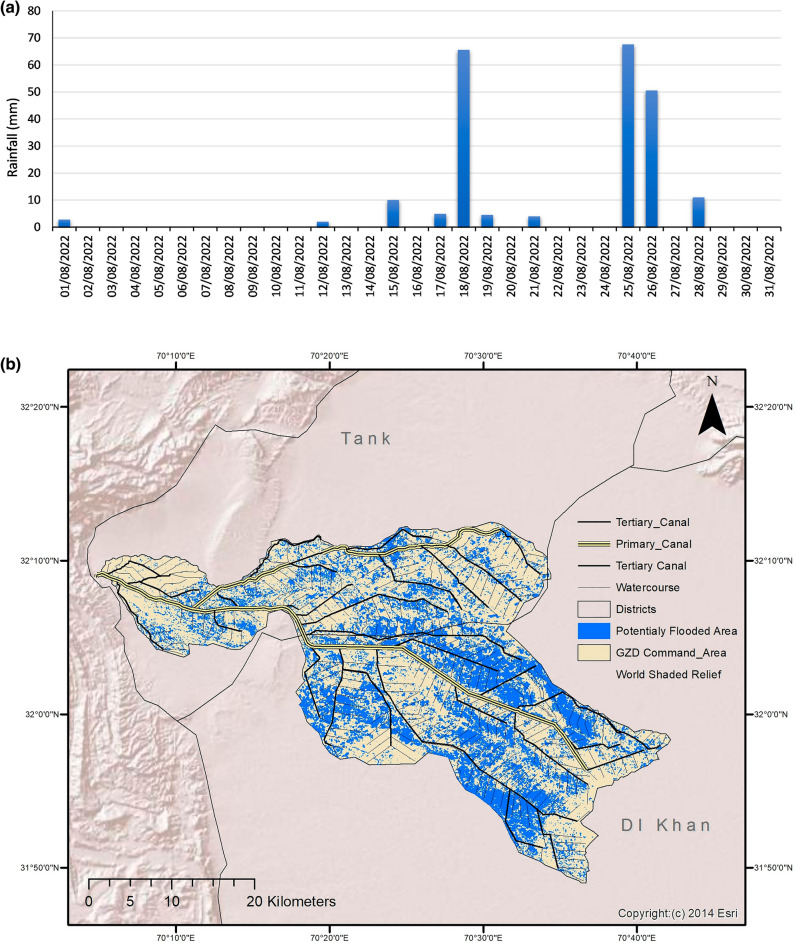
(a) Rainfall in DI Khan during the month of August 2022; (**b**) Flood inundation map of GZDCA based on Sentinal-1 imagery of 29 Aug 2022 using modified Google Earth Engine code developed by United Nation-Spider. The map has been derived automatically without validation data. Basemap credit: Copyright:(c) 2014 Esri, World Shaded Relief.

Table 2 shows a much lower number of rainy days of high intensity expected in the 2020s compared to the baseline. This is because of the mean value taken throughout 2011–2040. The projected numbers of days with intense rainfall for the year 2022 were 1 day each with rainfall > 38.1 mm and 50.8 mm. However, three intense rainfall events have been witnessed in GZDCA during August 2022 (see Fig. 3a). The intensity of rainfall is not exceptionally high, but it has created devastating consequences as flood inundation of around 33% of GZDCA as shown in Fig. 3b. The flooding has resulted in the loss of human lives, livestock, houses, and communication, irrigation, and agricultural infrastructure. The exact amount of flood losses is currently not available, however preeminently estimates of local authorities show 3900 affected houses, 20 people lost their lives and damages worth PKR 10 billion (USD 46 M) in GZDCA^89^.

### Heatwaves

Table 3 provides an alarming outlook of heatwaves in all future time slices. The number of hot days and heatwaves is showing an increasing trend with future time. Based on projected data in the 75th percentile, the number of hot days would be 4.5 times higher than the baseline whereas 5.3 times more heatwaves than the baseline are expected. Other heatwaves’ characteristics such as severity and length are also alarmingly higher than the baseline using data for the 75th and 90th percentiles. Similar results were reported by González et al.^9^ for New York City where they used data for 26 GCMs for projection and compared with a baseline 1971–2000.

**Table 3:** Features of heatwaves based on the projected mean daily temperature compared to the observed during the baseline period.

Time slices	Percentile	Number of hot days	Severity* of hot days (^°^C)	Number of heatwaves	Total length of heatwaves (days)	Longest heatwave (days)	Severity* of heatwaves (^°^C)
Baseline (1980–2005)		18	16.77	4	15	7	15.39
2020s (2011–2040)	10th	0	0.08	0	0	0	0.07
	25th	6	3.18	1	5	3	2.83
	75th	82	130.27	6	80	53	129.49
	90th	116	263.86	5	113	90	263.15
2050s (2041–2070)	10th	3	1.77	1	2	2	1.48
	25th	21	18.92	4	19	10	18.34
	75th	113	266.65	4	111	91	266.17
	90th	139	449.48	4	138	120	448.87
2080s (2071–2100)	10th	5	3.73	1	4	3	3.23
	25th	31	35.55	5	28	14	34.57
	75th	129	409.47	4	128	113	408.93
	90th	161	668.82	4	160	144	668.21

*Severity has been defined as the amount of mean temperature above the threshold: For example, a heatwave including 3 days with mean daily temperatures equal to 35, 36, 37 °C and a threshold equal to 30 °C. The Severity is calculated as (35−30) + (36−30) + (37−30) = 5 + 6 + 7 = 18 °C.

**Figure 4:**
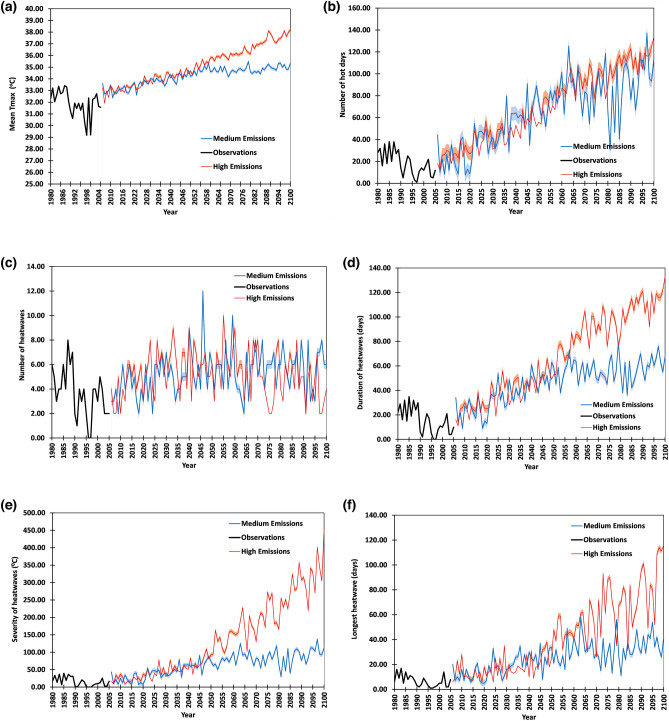
Results from bias-corrected projections for future assessments of (**a**) mean daily maximum temperature, (**b**) the number of hot days above the threshold, (**c**) number of heatwaves, (**d**) duration of heatwaves, (**e**) severity of heatwaves and (**f**) longest heatwave compared to the base period. Solid lines represent the multimodal mean of a 5 regional climate model ensemble, while shaded bands show 95% confidence intervals. Black lines indicate observations from two stations within GZDCA—DI Khan and Tank—between 1980 and 2005 as the base period.

To highlight the sensitivity of heatwave characteristics to medium emission and high-emission scenarios, the response is separately shown in Fig. 4a through f. The maximum temperature for GZDCA (Fig. 4a) shows far less deviation between the two emissions scenarios until 2060 when the deviation becomes pronounced. The number of hot days per year (Fig. 4b) shows an increasing trend but again the deviation between the two emission scenarios is not noticeable. The slope of the linear trendline by selecting an intercept equal to zero would show a slope of 0.97 for high emission and 0.93 for medium emission series. The number of heatwaves per year (Fig. 4c) for both emission scenarios does not show a clear trend. This is perhaps due to consecutive heatwaves coalescing into very long heatwaves, which becomes more likely as heatwaves increase in duration (Fig. 4d) and severity (Fig. 4e). The deviation between emission scenarios is visible in the case of heatwave duration and severity. It is also an artifact of the definition of heatwave used, which establishes an unchanging temperature threshold throughout the century based on the 95th percentile of the data from the baseline period. As mean temperatures increase, meeting the threshold on consecutive days becomes more likely. The logic of the inverse effect of long-duration (cumulative for each year) heatwaves on the number of heatwave events per year is supported by Fig. 4f which shows the results for the longest heatwave in each year and the trends of both emission scenarios profoundly resemble those of Fig. 4d.

### Drought indices and their relationship with wheat crop yield

Four selected drought indices (SPI, aSPI, RDI, and eRDI) are calculated using monthly temperature and precipitation data for DI Khan. The indices were calculated using observed data from 1980 to 2014 as input to DrinC software. The indices are calculated for various reference periods as mentioned in Table 4.

**Table 4:** Correlation coefficients (r) between annual crop yield loss and various drought indices using reference periods of 12, 5, 4, 3, 2, and 1 month.

Reference period	Months	SPI	aSPI	RDI	eRDI
12 months	Oct–Sep	0.30	0.31	0.31	0.32
5 months	Nov–Mar	*0.67*	*0.67*	*0.67*	*0.67*
4 months	Nov–Feb	0.61	0.60	0.61	0.60
3 months	Nov–Jan	0.24	0.24	0.25	0.24
2 months	Nov–Dec	0.13	0.13	0.14	0.14
1 month	Nov	0.00	0.00	0.01	0.01
	Dec	0.05	0.05	0.06	0.05
	Jan	0.53	0.53	0.55	0.54
	Feb	0.13	0.13	0.13	0.13
	Mar	0.43	0.43	0.44	0.44

*SPI* standardized precipitation index, *aSPI* agricultural-SPI, *RDI* reconnaissance drought index, *eRDI* effective-RDI.

*Italics* indicate the best correlation for the corresponding case.

The AquaCrop model is run at the annual time step for wheat using input data and setting as described in the Crop Modeling section. The same temperature and precipitation data are used as input to AquaCrop as used for the calculation of drought indices. Except for the temperature and precipitation, all other inputs to AquaCrop remained constant during annual runs, including surface water supply through the irrigation system. This can be justified because the irrigation system in GZDCA is fed from a reservoir behind a dam. The canal irrigation system downstream is constrained by design to supply a certain volume of water and any enhancement of the carrying capacity of the system is highly unlikely in the future. Any variation in Gomal River supplies in the upper catchment would only impact the storage in the reservoir and not the water supply to the fields in the command areas.

The simulated and observed yield correlate well (R^2^ = 0.65, n = 12 years) and show broadly similar trends. A such comparison should not be considered as a yardstick to gauge the efficiency of the model because of the limitations of both datasets. The average yield reported by the government agency involves sampling and aggregation. Therefore, the sample size, location, and size of the sampled plots play an important role in determining the average yield in a large area. Moreover, factors other than weather, soil, and water supply also play important roles in determining yield in a particular field plot. These factors include farmers' choices of seed variety, land preparation, fertilizer, pesticide, and timing and method to irrigate the field. As a combined effect, the yield in a plot can be much different from that in other fields around. This heterogeneity in yield can be reflected in the average yield computation of a randomly sampled large area. On the other hand, the aforementioned farmers’ choices cannot be incorporated precisely into the simulation model and the inputs to the model also involve many approximations and aggregations for the entire study area.

The results of the AquaCrop model include water productivity in kg(yield)/m^3^ evapotranspired, biomass, and dry yield. This paper has used annual yield loss (calculated using Eq. (2)) as an indicator to analyze how well different drought indices correlate with crop yield. The correlation coefficients are then calculated by comparing the annual time series of yield loss and selected drought indices for the period of (1980–2014). Selected drought indices for each year are separately calculated at different reference periods as shown in Table 4 to compute correlation coefficients.

The results show that the highest correlation coefficient is 0.67 when a reference period of 5 months (Nov–Mar) is used to calculate drought indices. This reference period exhibits the entire wheat crop length in GZDCA. The reference periods were then reduced to 4, 3, and 2 months and then by individual months to analyze sensitivity. Based on the correlation coefficient, January is the most critical month concerning crop life span when weather conditions play a crucial role. The weakest correlation coefficient is for Nov and Dec when the crop is sown and at a very early phenological stage. The indices calculated for the entire year also give a weak correlation.

Table 4 also reveals that all four selected drought indices have correlated with yield loss quite similarly. For example, the correlation coefficient when a 5-month reference period is selected is the same for all drought indices. Overall RDI and eRDI have a stronger correlation with yield loss than SPI and aSPI. There is no significant difference between correlation coefficients for RDI and eRDI which makes it insensitive to use any of these drought indices. However, the slight advantage of eRDI over RDI is that it incorporates the substitution of total precipitation by effective precipitation and includes potential evapotranspiration in the calculation. For a couple of instances, the drought indices in their standard form correlate better with annual yield loss than their agricultural variant e.g., for a 4-month reference period both SPI and RDI show a better correlation than aSPI and eRDI, for 1-month (Jan) RDI show better correlation than eRDI. These results corroborate the findings of Tigkas et al.^65^ who calculate SPI and aSPI in parts of Greece and find correlation coefficients with an annual yield loss of winter wheat. In a similar study^78^ they calculated correlation coefficients for RDI and eRDI. In our study, we have compared four drought indices by correlating them with the annual yield loss. The wheat crop characteristics in Greece and the study area are not the same and hence the range of correlation coefficients also differ.

Because drought conditions fluctuate naturally, it is helpful to look at expected drought prevalence over several years in the future to explore how it is connected to climate change. The correlation coefficient between drought indices and yield loss suggests that for drought impact studies on agriculture, the drought indices should be calculated for specific reference periods in connection with local crops. Particularly when biased corrected future data is used for calculating drought indices it becomes more imperative to pick a reference period separately for each local crop. Simply selecting a drought index for a 12-month reference period may give a misleading outlook.

Chen et al.^90^ have used a similar approach to establish the relationship between simulated winter yields (using the DSSAT-CERES-Wheat model) at 108 sites in China and SPEI at various timescales. Chen et al.^90^ found a strong correlation between 4-month SPEI and yield which is affected by drought conditions during the jointing to milk stages of winter wheat. These results corroborate the findings of our analysis.

The physical mechanism of drought occurrence in Pakistan is of more importance to meteorologists and the scientific community. The positive phases of circumglobal teleconnection and Summer North Atlantic Oscillation are related to excessive rainfall during the summer season over Pakistan. The water vapor transportation (Arabian Sea to South Asia) associated with the monsoon trough, is critical for dry and wet conditions over Pakistan during summer season^91,92^. On the other hand, Niño4, sea surface temperature, and multivariate El Niño-Southern Oscillation (ENSO4.0) Index are the most influential factors for seasonal droughts across Pakistan^93^.

The results presented in this section are corroborated by the findings of recent studies on climate change projections and the socioeconomic impact of heatwaves and droughts in South Asia^19,20^ suggesting that the frequency of 50-year historical droughts might double across 80% of South Asian land area under 1.5 °C warming. The dissection of results on a sub-regional scale reveals that population exposure to extreme heat events is highest in southeastern India and southern Pakistan accounting for more than 75% of the total exposure for SA. The study area in this paper is located in the southern part of Pakistan where the population is highly vulnerable to exposure to heat events.

### Agricultural drought outlook

**Figure 5:**
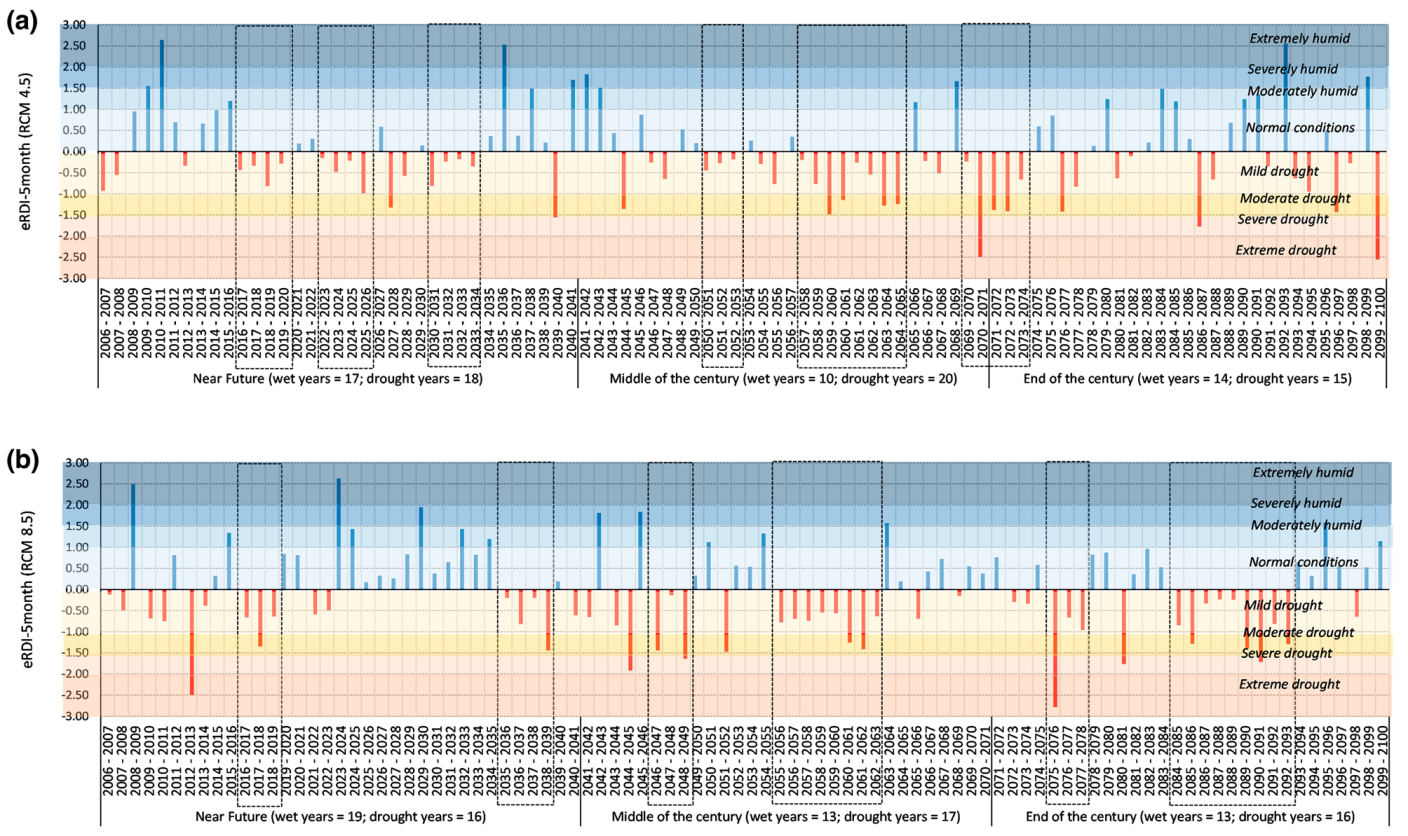
Effective Reconnaissance Drought Index (eRDI) for DIKhan. The calculation is based on predicted data of minimum and maximum temperature and precipitation, calculated for a reference period of five months (Nov–Mar) corresponding to the wheat crop season.

**Figure 6:**
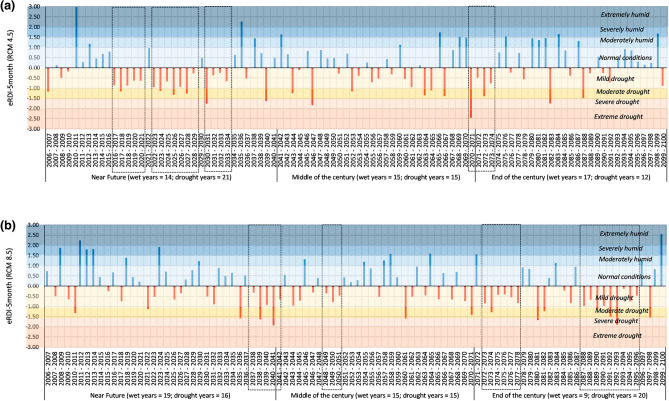
Effective Reconnaissance Drought Index (eRDI) for Tank. The calculation is based on predicted data of minimum and maximum temperature and precipitation, calculated for a reference period of five months (Nov–Mar) corresponding to the wheat crop season.

Figures 5 and 6 show 5-month eRDI using biased corrected temperature and precipitation data for DI Khan and Tank districts respectively. The results are separately presented for the two emission scenarios to highlight the sensitivity of drought indices to emission scenarios 4.5 and 8.5. The plot area is shaded in colored bands in Figs. 5 and 6 to highlight the subjective interpretation of drought indices. Also, summary statistics i.e., the number of dry and wet years in each future time slice are shown below the abscissa. We have used dashed rectangles to show long-term drought conditions defined here as the instances where three or more consecutive years would be experiencing agricultural (wheat crop) drought conditions based on eRDI-5 month.

Figure 5 shows that in DI Khan 53 agricultural drought years and 6 long-term agricultural drought conditions are expected in the twenty-first century under emission scenario 4.5. On the other hand, a slightly lesser number of drought years (49) are expected in the case of emission scenario 8.5 which seems counterinitiative. However, a closer look shows that the magnitude of drought is much severer in the case of emission scenario 8.5 than 4.5. There are more years with moderate drought or beyond under emission scenario 8.5 than 4.5.

Figure 6 shows that in Tank, the expected agricultural drought years would be 51 and 49 under emission scenarios 8.5 and 4.5 respectively. Moreover, emission scenario 8.5 exhibits more long-term agricultural drought conditions, particularly in the middle and end of the century. Contrarily the timing of long term-drought conditions is in the near future when emission scenario 4.5 is considered.

Overall, the outlook of drought for DI Khan and Tank are similar in terms of summary statistics which is understandable as both cities have quite comparable climate conditions.

## Conclusions

This article focused on an underrepresented agricultural area in Pakistan for climate projections and its associated hazards. The analysis covers calculating indices and metrics of potential climate-related hazards (e.g., heatwaves, precipitation extremes, and droughts) that can result in negative outcomes for the inhabitants, infrastructure, and agriculture in GZDCA.

The GZDCA is a representative sample of typical agricultural areas in South Asia where irrigation schemes are developed. The agriculture system and hence the food production is highly vulnerable to climate hazards. Particularly, heavy rainfall during monsoon (July-Sep) triggers floods that damage the summer crops (rice, sugarcane, cotton). The GZDCA witnessed heavy rainfall in August 2022 causing one-third of the area to be affected by the flood. The winter crops notably wheat is more vulnerable to droughts and heatwaves. The results of the AquaCrop model using observed climatic data (1980–2005) have shown the annual response of wheat yield cultivated in GZDCA. The yield response correlates quite well with the agricultural drought indices, especially eRDI. Hence eRDI is the best-suited agricultural drought index among other options considered in this study. The results suggest that the magnitude of eRDI correlates better with wheat yield response when climate data for 5 months (Nov–Mar) was used as input for drought indices and the crop model. The drought indices are commonly calculated based on an annual basis (using 12-month climatic data) which may provide a broader outlook but does not explain agricultural drought characterization for specific crops. Therefore, agricultural drought indices should be calculated separately for major crops using a similar approach used in this article, particularly when calculated with projected climatic data and intended to be used for planning and preparedness. The results of eRDI-5 months for GZDCA show the likelihood of long-term (3 or more continuous drought years) agricultural drought in the near future. This means that wheat production is highly vulnerable to drought and requires careful plans to face this challenge to food production.

The heatwave characteristics and agricultural drought indices for the future show that the GZDCA is expecting frequent climate stresses. The results of this study would be of particular interest to the district administration, and the government agencies for disaster management, agriculture, and livestock. In Pakistan, the district administration usually steers the disaster preparedness efforts and plays a coordination role amongst various agencies including agriculture and livestock.

A policy recommendation from this study is that the climate risk assessments should be done at a district scale that can focus on climate hazards having more potential to affect human lives and civil infrastructure. At the same time, a rather pragmatic approach could be to consider agricultural areas (that do not necessarily follow administrative boundaries of districts) for climate risk assessments with a focus on climate hazards affecting agricultural systems. The insights from GZDCA as a focused area in this study support this argument. The National Climate Change Policy of Pakistan^94^ emphasizes disaster preparedness on a national scale however probably a more efficient way to accomplish this is to delegate it to the scale of districts/agricultural areas. This would improve the planning process when based on local assessments of climate risks that are more focused and context specific. Future research can consider more crops and corresponding reference periods to calculate agricultural drought indices.

This study has only used RCMs outputs from CORDEX-South Asia frameworks and has not considered outputs from other models. A limitation of this study is the inbuilt assumption of statistical downscaling approaches that the relationships between the large-scale and local variables do not change in the future– this assumption of statistical stationarity may not remain valid in a nonstationary/changing climate^95^. At the time of analysis presented in this paper was completed, the data of required spatial and temporal resolution from Coupled Model Inter-comparison Project Phase six (CMIP6) was not widely accessible. It is therefore suggested that future studies should use the latest datasets and choose appropriate Shared Socioeconomic Pathways (SSPs)^96^ developed by the climate research community. The authors suggest for future studies using SSPs for climate projections and calculating eRDI for specific crops in the agricultural area of interest.

## Data Availability

The datasets analyzed during the current study are not publicly available by the collecting agency in Pakistan but are available from the corresponding author on reasonable request.
